# Using Micro-Electrode-Array Recordings and Retinal Disease Models to Elucidate Visual Functions: Simultaneous Recording of Local Electroretinograms and Ganglion Cell Action Potentials Reveals the Origin of Retinal Oscillatory Potentials

**DOI:** 10.3390/bioengineering10060725

**Published:** 2023-06-15

**Authors:** Wadood Haq, Eberhart Zrenner, Marius Ueffing, François Paquet-Durand

**Affiliations:** Centre for Ophthalmology, Institute for Ophthalmic Research, Elfriede-Aulhorn-Straße 7, 72076 Tuebingen, Germany; ez@uni-tuebingen.de (E.Z.); marius.ueffing@uni-tuebingen.de (M.U.); francois.paquet-durand@uni-tuebingen.de (F.P.-D.)

**Keywords:** ERG, micro-ERG, oscillatory potentials, multi-electrode recordings, retinal degeneration, RD models, 3R principles

## Abstract

Background: The electroretinogram (ERG) is an essential diagnostic tool for visual function, both in clinical and research settings. Here, we establish an advanced in vitro approach to assess cell-type-specific ERG signal components. Methods: Retinal explant cultures, maintained under entirely controlled conditions, were derived from wild-type mice and *rd10 rod-* and *cpfl1* cone-degeneration mouse models. Local micro-ERG (µERG) and simultaneous ganglion cell (GC) recordings were obtained from the retinal explants using multi-electrode arrays. Band-pass filtering was employed to distinguish photoreceptor, bipolar cell, amacrine cell (AC), and GC responses. Results: Scotopic and photopic stimulation discriminated between rod and cone responses in wild-type and mutant retina. The 25 kHz sampling rate allowed the visualization of oscillatory potentials (OPs) in extraordinary detail, revealing temporal correlations between OPs and GC responses. Pharmacological isolation of different retinal circuits found that OPs are generated by inner retinal AC electrical synapses. Importantly, this AC activity helped synchronise GC activity. Conclusion: Our µERG protocol simultaneously records the light-dependent activities of the first-, second-, and third-order neurons within the native neuronal circuitry, providing unprecedented insights into retinal physiology and pathophysiology. This method now also enables complete in vitro retinal function testing of therapeutic interventions, providing critical guidance for later in vivo investigations.

## 1. Introduction

The electroretinogram (ERG) is the gold standard for non-invasive investigations into the eye’s visual function. It is a standardised non-invasive diagnostic method in humans [1,2] that assesses the retina’s electrical response to light [3,4,5]. The use of appropriate stimulus conditions allows access to either rod- or cone-derived activity, which is crucial when investigating retinal diseases [6,7,8,9,10]. Mouse ERG data are generally comparable to those of humans [5] and can thus link clinical findings in many retinal disorders with basic research in search of new treatment strategies (e.g., genetic treatments [6], drug treatments [11,12]).

Due to its relevance to research and clinical applications, the components of the ERG signals originating from and shaped by distinctive types of cells have been intensively investigated in previous research [5,7,13]. In brief, the first component of the ERG, the so-called a-wave, originates in the photoreceptors and is identified by its characteristic negative deflection after the onset of light [14,15,16,17,18]. The negative polarity (hyperpolarisation) of the a-wave results from the light-induced closure of cation channels along the photoreceptor outer segments [14,19,20]. Rod and cone photoreceptors differ in their light sensitivity and response kinetics. Accordingly, the highly light-sensitive rod responses are investigated after full dark adaptation, whereas cone responses are commonly recorded using background lighting to saturate rod photoreceptors [21,22]. The second main component of the ERG is the b-wave, which is a positive deflection associated with the activity of depolarising bipolar cells (BC) [4,13,23,24,25]. In addition to the neuroretinal a- and b-wave responses, glial and retinal pigment epithelial cell activity is also registered in the ERG signal as the c-wave, which is a slow positive-deflection signal following the b-wave [26,27,28]. Since the retinal explants used in the present study consist only of a thin layer of retinal pigment epithelial cells without attachment to the choroid, the c-wave response will not be further considered in this article.

Oscillatory potentials (OPs) appear as a group of waves superimposed on the b-wave [29,30,31]. They are an important component of the ERG [32,33,34] and have been used in diagnosing a variety of eye diseases, including elevated intraocular pressure [35,36], longstanding systemic hypertension, central retinal vascular occlusion, retinopathy of prematurity, different types of retinal dystrophy and degeneration, and even retinal toxicity [32,37,38,39]. Moreover, it has been suggested that OPs are a useful indicator for early diagnosis of diabetic retinopathy since human and animal studies indicate a diabetic retinopathy-dependent reduction in OP amplitudes [32,37,38,39]. Despite their relevance to research and clinical applications, the origin of OPs has not yet been definitively determined. Alternative hypotheses propose their generation in the outer retina (rod–cone coupling) [40] or the inter retina through neural interactions between BCs, amacrine cells (ACs), and ganglion cells (GCs) [29,30,31,41].

We aimed to dissect the integrated ERG signal into its cell-type-specific components. Moreover, we investigated the unresolved role of inner retinal connectivity in generating and shaping OPs in the ERG. Since assessing the functions of individual cells in the inner retina is technically challenging in living animals, we used an advanced in vitro approach based on retinal explants: (1) Based on a methodology originally developed by Stett and colleagues [42], we established an advanced setup and method for multi-electrode array (MEA) recordings. Our MEA consisted of 59 micro-electrodes, each with a 30 µm diameter and a spacing of 200 µm, enabling us to assess the light-evoked responses originating from different layers of the retinal tissue. These local in vitro field potentials, recorded within the micro-metre range at each micro-electrode of the MEA, reflected the typical characteristics of the in vivo retinal ERG [42,43,44]. We refer to these recordings as “micro-ERG” (µERG). Moreover, GC activity (spikes) was recorded simultaneously and directly correlated with the diverse components of the µERG. A high data sample rate of 25 kHz and broad filter ranges allowed us to obtain exceptionally detailed µERG recordings. (2) To discriminate rod and cone photoreceptor responses, scotopic and photopic µERG protocols were established (adapted from [21,22]). These protocols were validated using mouse mutants that lacked either rod function (i.e., the *rd10* mouse [45,46]) or no cone function (i.e., the *cpfl1* mouse [47]) [21,22,48]. Additionally, our approach recorded GC activity (spikes) and correlated it with the scotopic and photopic photoreceptor responses for each light intensity. (3) Highly specific drug treatments were employed to investigate the origin of different µERG components in the inner retina.

Here, we report an advanced µERG recording method with superior temporal and spatial resolution that enables the simultaneous capture of the electrophysiological activity of diverse outer and inner retinal cell types. We employed this new experimental setup in conjunction with well-validated drugs to conclusively demonstrate that OPs are generated by ACs in the inner retina. This example illustrates the strength and versatility of the µERG technique and highlights it as an essential new complement to conventional electrophysiological techniques.

## 2. Materials and Methods

### 2.1. Animals

This study used 15 healthy Bl6 wild-type mice (C57BL/6J), 5 *cpfl1* mice (B6.CXB1-*Pde6c^cpfl^*^1*/J*^) with cone function loss, and 5 *rd10* mice (C57BL/6J-*Pde6b^rd^*^10^) with rod dystrophy, ranging in age from 4 to 5 weeks. The mice were housed under standard white cyclic lighting and had free access to water and food. Before the experiments, the mice were kept for 12 h in an air-ventilated, light-tight box for dark adaption. The animals were anesthetised in a carbon dioxide atmosphere and were immediately sacrificed by cervical dislocation.

### 2.2. Tissue Preparation

All the preparation and handling procedures were carried out under dim red-light conditions. The eyes were enucleated immediately after euthanasia. The incubation, dissection, and recording medium used was carbonated (95% CO_2_/5% O_2_) artificial cerebrospinal fluid (ACSF) containing the following concentrations (in mM): 125 NaCl, 26 NaHCO_3_, 2.5 KCl, 2 CaCl_2_, 1.25 NaH_2_PO_4_, 1 MgCl_2_, and 20 glucose (pH 7.4–8.0). All chemicals were obtained from Sigma-Aldrich (Darmstadt, Germany). The eyes were hemisected, and the lens and vitreous were carefully removed. To minimise damage, the forceps only touched the circumference of the retina, not the area of the retina considered for recording.

### 2.3. Retinal Recordings

To access the light-dependent retinal responses (photoreceptors µERG and GC spikes), electrophysiological recordings were performed using an MEA system (USB-MEA60-Up-BC-System-E from Multi Channel Systems (MCS), Reutlingen, Germany) equipped with an MEA 200/30iR-ITO-pr (59 recording electrodes; 30 µm diameter; 200 µm spacing). The average impedance of the MEA electrodes was 0.7 MOhm (nanoZ v 1.2, MCS, Reutlingen, Germany). For recording, the retina was placed in the MEA recording chamber with the GC side facing down onto the electrode field. The retina rested in darkness on the MEA for at least 45–60 min before the execution of the light-stimulation protocol. During the measurements, the MEA chamber was continuously perfused at a rate of 2 mL/min with carboxygenated ACSF and maintained at a temperature of ~32 °C. To collect unfiltered raw data, the recordings were performed at a 25,000 Hz sampling rate. The recording protocol was set within the MC_Rack software (v 4.6.2, MCS), and the attached digital I/O box (MCS) was utilized to realize the trigger-synchronised operation of the light stimulation (LEDD1B T-Cube, Thorlabs, Dachau Germany) and MEA recording.

### 2.4. Light Stimulation

**Figure 1:**
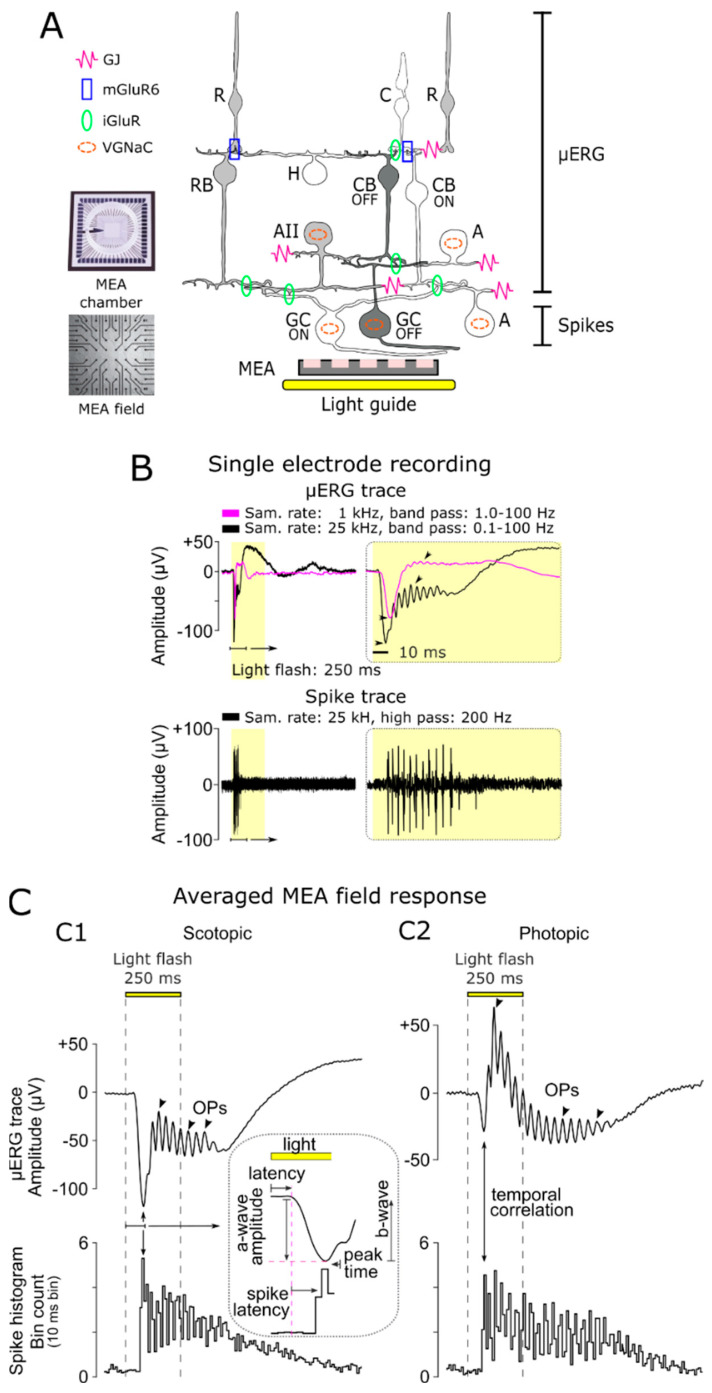
Multi-layered recordings of light-evoked responses in the retina. (**A**) Experimental setup: Retinal explants are placed in the multi-electrode array (MEA) chamber with ganglion cells (GCs) contacting the electrode field (59 electrodes—electrode diameter: 30 µm; spacing: 200 µm). Light flashes of different intensities were applied through the transparent MEA via a light guide and collimator (250 ms full-field flash). Sketch of retinal circuitry showing functional pathways that govern the micro-electroretinogram (µERG) response: rod (R) and cone (C) photoreceptor, horizontal cell (H), rod bipolar cell (RB), ON- and OFF-type cone bipolar cells (CB), amacrine cell (AII) relaying the rod system (grey) on to the cone system (white), representative diverse amacrine cells (A) modulating retinal functions, and ON (white)- and OFF (dark)-type ganglion cells (GC). µERG relevant functional connections: gap junctions (GJ), metabotropic glutamate receptors (mGluR6), inotropic glutamate receptors (iGluR), and cells with voltage-gated sodium channels (VGNaC). (**B**) Differentiation of multi-layer retinal field potentials (representative single-electrode recording): µERG, signal of rod and cone photoreceptors, BCs (magenta: sample rate 1 kHz, band-pass filtered 1–100 Hz; black: sample rate 25 kHz, band-pass filtered: 0.01–100 Hz), and GC spike responses (sample rate: 25 kHz; high-pass filter: 200 Hz). The high sampled data revealed larger amplitude and signal details (arrowhead). (**C**) Representative averaged MEA field responses under (**C1**) scotopic (12 h dark-adapted; flash intensity: 4.20 × 10^13^ photons/cm^2^/s) and (**C2**) photopic (5 min light-adapted at background light intensity of 4.20 × 10^13^ photons/cm^2^/s; flash intensity: 4.20 × 10^15^ photons/cm^2^/s) conditions: µERG traces (upper panel), oscillatory potentials (OPs, arrowhead), and corresponding spike responses (spike histogram, 10 ms bin spike count; lower panel). The inset shows a magnified µERG trace and illustrates the latencies between a-wave, b-wave, and GC spiking activity.

Light stimulation (white light LED, 2350 mW, MCWHD3, Thorlabs, Dachau, Germany) was applied from beneath the MEA (transparent glass) guided by optic fibre and optics (collimated full field, Figure 1A). The calibration of the applied light-stimulation intensities was performed using the USB4000-UV-VIS-ES spectrometer (Ocean Optics, Orlando, FL, USA). To discriminate the rod photoreceptor and cone photoreceptor responses, a µERG light-stimulation protocol for ex vivo MEA recordings was established, which was adapted from standardised in vivo ERG protocols [21,22]. For the scotopic recordings (rod photoreceptor responses), the mice were dark-adapted (in vivo > 12 h), whereas for the photopic recordings (cone photoreceptor responses), the retinal explants were light-adapted on the MEA (ex vivo, 5 min at an intensity of 4.20 × 10^13^ photons/cm^2^/s), subsequent to scotopic stimulation. The parameters of the light stimuli were set as a light flash of 250 ms long (3 repetitions per light intensity, 20 s intervals). Neutral density filters mounted in a filter wheel were used to set the light intensity (filter wheel: FW212CWNEB; filter: AR Coated, 350–700 nm, optical density: 0.5–5.0, Thorlabs, Germany). To monitor the proper application of the light-stimulation protocol, the signal of a photodiode mounted in the vicinity of the LED was recorded simultaneously (MCRack, MCS, Reutlingen, Germany). For the scotopic condition, there were 11 stimuli in 0.5 log steps ranging from −5.0 to 0.0 (1.33 × 10^9^ to 4.20 × 10^13^ photons/cm^2^/s), where a 0.0 intensity corresponded to the illumination for photopic incubation and the background illumination during the photopic recordings. For the photopic condition (background light: 4.20 × 10^13^ photons/cm^2^/s), there were four stimuli in 0.5 log steps ranging from +0.5 to +2.0 (1.33 × 10^14^ to 4.20 × 10^15^ photons/cm^2^/s).

### 2.5. Pharmacology

The pharmaceuticals were bath-applied for 30 min of incubation before the light stimulation was executed. We applied (in µM) 20 NBQX (AMPA/kainate-type GluR antagonist; 2,3-Dioxo-6-nitro-1,2,3,4-tetrahydro-benzo[f] quinoxaline-7-sulfonamide) and 100 L-AP4 (mGluR6 agonist; L-2-amino-4-phosphonobutyric acid) obtained from Bio Trend, 50 carbenoxolone (CBX, gap-junction blocker (3β,20β)-3-(3-Carboxy-1-oxopropoxy)-11-oxoolean-12-en-29-oic acid disodium) obtained from Sigma-Aldrich, and 3 tetrodotoxin (TTX, voltage-gated sodium channel blocker) obtained from Sigma-Aldrich (Darmstadt, Germany).

### 2.6. Data Analysis and Statistics

The raw data files of the MEA recording were filtered using the Butterworth second order (MC_Rack, MCS, Reutlingen, Germany) to distinguish the µERG (field potentials: band passes 0.01–100 Hz) and spikes (high pass 200 Hz). To process the recorded data within MATLAB (R2019a, MathWorks, Natick, MA, USA), the filtered MC_Rack files were converted to *.hdf files using MC DataManager (v1.6.1.0). The software bundle McsMatlabDataTools (MCS, Reutlingen, Germany) enabled the import of the HDF5 files into MATLAB. Further data processing was implemented using custom-written MATLAB scripts. Spike detection was performed at a signal threshold of ±17 µV, considering only bimodal traces and eliminating multiple detections of cells on different electrodes [49]. The trigger timestamps of the stimuli were used to detect the light-induced retinal responses. A time binning of 10 ms was used for the spike histogram (overall MEA electrodes) to account for early-responding ON and delayed-responding OFF cells, covering the ON–OFF cells [49]. The displayed µERG traces represent an average of the MEA recording field for each condition. For the analysis of the OPs, the µERG signals were band-pass filtered (10–100) to remove the a-wave deflection [50]. Statistical significance was estimated using one-way ANOVA followed by Dunnett’s multiple comparisons test (*p* < 0.001, *p* < 0.1, and *p* < 0.5). Experimental data are given as mean ± SEM. Figures were prepared using Inkscape (inkscape.org, v1.2, accessed on 6 June 2022).

## 3. Results

### 3.1. Characteristics of Light-Evoked Responses in Retinal Explants

To assess the light-evoked multi-layer responses of retinal explants, we performed multi-electrode array (MEA) recordings (Figure 1A). A total of 59 MEA electrodes (each with a 30 µm diameter and a spacing of 200 µm spacing) were used to record the local light-evoked retinal activity. The recorded raw data, originating from different retinal layers, were filtered to distinguish the field potentials (µERG, photoreceptor, BC responses, and OPs) from the GC action potentials (spikes; Figure 1A,B). The GC responses were isolated via a high-pass filter (Figure 1B, lower panel: 200 Hz, Butterworth, second order [49]), and the µERG signal was obtained through low-pass filtering (Figure 1B, upper panel). The higher data sample rate (25 kHz) and broader band-pass filter (0.1–100 Hz) revealed signal details associated with the inner retina that have not been seen in previous studies working with lower resolutions (e.g., at 1 kHz sampling and 10–100 Hz band pass [42,44]).

### 3.2. Differentiation of Cellular Origin of Neuroretinal Signals

To distinguish the rod and cone responses, MEA recordings were conducted from retinal explants under scotopic (dark-adapted) and photopic (light-adapted) conditions (Figure 1C). The scotopic and photopic in vitro µERG signals corresponded to the neuroretinal response characteristics of ERGs obtained from living animals. However, it is worth noting that the a-wave of the cone response under photopic conditions was up to threefold smaller than that of rods under scotopic conditions because only 3–5% of the photoreceptors in the mouse retina are cones [51].

To investigate the cellular origins of different µERG signal components, we compared the temporal development of the µERG signal with GC spiking activity. The first negative deflection, the a-wave, indicated the light-dependent hyperpolarisation of the photoreceptors. This started with a low latency after the onset of the light flash (scotopic at strongest intensity: 46.12 ± 0.58 ms; photopic at strongest intensity: 56.36 ± 0.93 ms; Figure 1C, inset). Accordingly, the GC responses occurred with additional latency (scotopic: 32.60 ± 2.66 ms; photopic: 18.87 ± 2.02 ms) and correlated temporally with the peak of the a-wave, indicating a network-mediated processing delay. After the a-wave peak (scotopic: 78.72 ± 3.24 ms, photopic: 75.22 ± 2.95 ms), the b-wave deflection correlated with the onset of GC spiking activity. Superimposed on the b-wave were OPs, which were evident under both scotopic and photopic conditions. Remarkably, the oscillations of GC spiking activity correlated in time with the duration of OPs (Figure 1C), indicating a common modulator.

### 3.3. Scotopic and Photopic µERG Protocol for Retinal Explants

**Figure 2:**
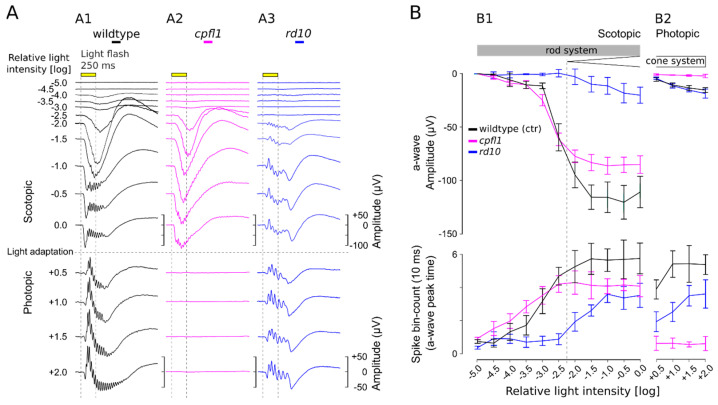
µERG recordings on rod and cone mutant mouse models. (**A**) Representative µERG recordings of (**A1**) wild-type mice, (**A2**) the cone photoreceptor function loss-1 (*cpfl1*), and (**A3**) the retinal degeneration-10 (*rd10*) mouse models. The µERG protocol included eleven 0.5 log unit steps with increasing light intensities (upper panel; from 1.33 × 10^9^ to 4.20 × 10^13^ photons/cm^2^/s) and four photopic stimuli in 0.5 log steps (lower panel; from 1.33 × 10^14^ to 4.20 × 10^15^ photons/cm^2^/s). The light intensity is presented in log units relative to the light intensity required for photopic adaptation (5 min; 4.20 × 10^13^ photons/cm^2^/s) and also serves as the background light. (**B**) Evaluation of correlated photoreceptor and ganglion cell light responses. Analysis of photoreceptor a-wave responses (µV; upper panel) and ganglion cell spike counts (10 ms bin; lower panel) in retinal µERG recordings for wild-type, *cpfl1*, and *rd10* retinas (*n* = 5). (**B1**) Scotopic conditions illustrating rod system activity. (**B2**) Photopic conditions for cone system activity. At −2.0 log units, the boundary between rod-only and mixed rod–cone responses is indicated (dashed line). Error bars indicate mean ± SEM.

**Figure 3:**
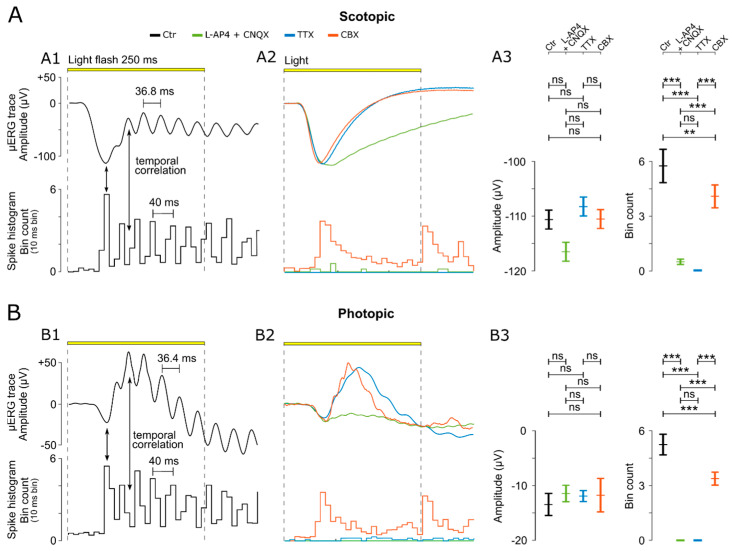
Analysis and experimental manipulation of µERG oscillatory potentials. (**A**) Analysis of µERG data under scotopic conditions. (**A1**) µERG and ganglion cell (GC) responses obtained from untreated (Ctr) wild-type retinal explants. Arrows indicate characteristic temporal correlations between the a-wave peak and GC spike response peak (cf. Figure 1, inset), as well as between the b-wave oscillatory potentials (OPs) and GC spiking activity. (**A2**) Representative µERG and GC responses recorded in the presence of either a combination of ionotropic glutamate receptor blocker CNQX and metabotropic glutamate receptor agonist L-AP4, the voltage-gated sodium channel blocker TTX, or the gap-junction blocker (CBX). (**A3**) Evaluation of µERG a-wave amplitude (µV) (left panel) and ganglion cell (GC) spike responses (counts per 10 ms bin) (right panel) under control and in the presence of respective blockers. (**B**) Analysis of µERG under photopic conditions (as in (**A**)). For each treatment condition, a separate wild-type retina was used (*n* = 5 retinas/recording; each recording averaged from 59 electrodes). Statistical significance was estimated by one-way analysis of variance (ANOVA), followed by Dunnett’s multiple comparisons test (***: *p* < 0.001; **: *p* < 0.1; and ns: not significant). Error bars indicate mean ± SEM.

Using wild-type mouse retina, we established an in vitro µERG protocol for retinal explants with light-stimulation intensities increasing in 0.5 log unit steps for scotopic (11 steps) and photopic (4 steps) conditions (Figure 2A). To analyse retinal signals, the amplitude of the a-wave was plotted against the concomitant GC spike responses (Figure 2B). Note that the maximum a-wave deflection corresponded with the GC spike response peak (cf. Figure 1C). No light responses were detected at the lowest light intensity used (−5.0 log unit = 1.33 × 10^9^ photons/cm^2^/s) (Figure 2A,B). The a-wave amplitudes and spike frequencies of the wild-type mouse increased almost linearly with the rising light intensity up to an intensity of −2.0 log units. Responses elicited with light stimuli less than −2.5 log units were considered pure rod responses, which displayed relatively small a-waves but robust GC responses. The recordings of the rod-deficient *rd10* mouse support this interpretation since no responses were seen up to −2.5 log units (Figure 2 and Figure 3, blue). At higher light intensities (≥−2.0 log units), retinal responses were larger, yet the amplitudes of both the a-wave and GC spike frequencies eventually saturated. The retinal responses obtained within this scotopic range were considered rod-dominant responses since ~97% of mouse photoreceptors are rods, whereas only ~3% are cones [51]. To isolate the cone responses, the retinal explant was light-adapted (photopic adaptation on MEA) to saturate the rods so that only cone signals were registered [21,22]. Due to the low number of cones, the photopic a-wave amplitude was reduced by ~93%, whereas GC spike frequencies were comparable to responses in the upper scotopic ranges. With increasing light-stimulation intensities, a minor but continuous increase in the cone a-wave amplitude was observed, while at the same time, the correlated GC spike responses appeared to be saturated (Figure 2B). The photopic retinal response was considered cone-dominant due to its characteristic shape with small a-waves and a pronounced b-wave with OPs. Moreover, the GC responses served as an additional indicator to verify light-correlated responses when the a-wave was very small, for example, at very low scotopic light intensities or in the low photopic range.

To validate the methodology and verify the responsiveness range of rods and cones, mouse mutants for retinal degeneration were recorded and verified (Figure 2; cone function loss: *cpfl1* (magenta); rod degeneration: *rd10* (blue)). In 4–5-week-old *cpfl1* mouse retina (Figure 2), rod responses were evident in the scotopic recordings (a-wave and spikes), whereas under photopic conditions, cone responses were completely absent, which is in accordance with previous *cpfl1* in vivo ERG findings [48]. In contrast, the *rd10* mouse retina at the same age (Figure 2) showed clear cone responses under photopic conditions (a-wave and spikes). However, under scotopic conditions, the first retinal responses were registered at light intensities above −2.0. These µERG responses most likely originate from cones since in 4–5-week-old *rd10* mice, rods are virtually absent or dysfunctional [45,46,52] and the characteristic cone-like shape of the µERG signals is visible (small a-wave but very pronounced b-wave with OPs). These *rd10* mouse µERG recordings are also in agreement with previous in vivo *rd10* ERG results [45,46].

### 3.4. OPs Originate in the ACs and Synchronize GC Activity

The scotopic and photopic recordings showed OPs superimposed on the b-wave. Surprisingly, the comparison with the simultaneously recorded GC activity revealed a correlated oscillation between the b-wave’s OPs and the spike histograms in the temporal domain (Figure 3(A1,B1)). The maximum GC response was found at the peak negative deflection of the a-wave, and the following spike responses appeared to be phase-shifted compared to the b-wave OPs. The oscillation frequency for the scotopic and photopic responses was estimated at 36.52 ± 2.99 Hz and 38.17 ± 2.62 Hz, with a peak-to-peak µERG time period of 36.51 ± 2.99 ms for the scotopic condition and 38.17 ± 2.62 ms for the photopic condition. The peak-to-peak time for the spike responses was estimated at 40 ms (note 10 ms time binning).

To isolate the ERG-relevant retinal signalling pathways, we selectively inactivated specific parts of the retinal circuitry using established and well-validated drugs (Figure 3(A2,B2)). First, we isolated the outer retinal responses (i.e., photoreceptors, a-wave) from the inner retinal responses to assess whether the cellular source synchronising the BC–GC activity was located in the inner retina (cf. Figure 1A). For this purpose, we used the ionotropic glutamate receptor (iGluR) antagonist CNQX combined with the metabotropic glutamate receptor (mGluR6) agonist L-AP4. The combination of these two drugs blocked the photoreceptor signal to both ON-BC and OFF-BC, respectively. These drugs also blocked the input from rod-BCs and cone-BCs to GCs. As a result, the b-wave, with its characteristic OPs and corresponding GC responses, disappeared while the photoreceptor a-wave responses remained normal (representative traces: Figure 3(A2,B2); statistical evaluation: Figure 3(A3,B3)). This result thus indicated an inner retinal origin of OPs. Remarkably, the absence of a b-wave led to a somewhat larger negative deflection of the a-wave (Ctr: −110.62 ± 14.68; L-AP4 and CNQX: −116.52 ± 13.98), supporting the ‘push-pull’ model [53].

**Figure 4:**
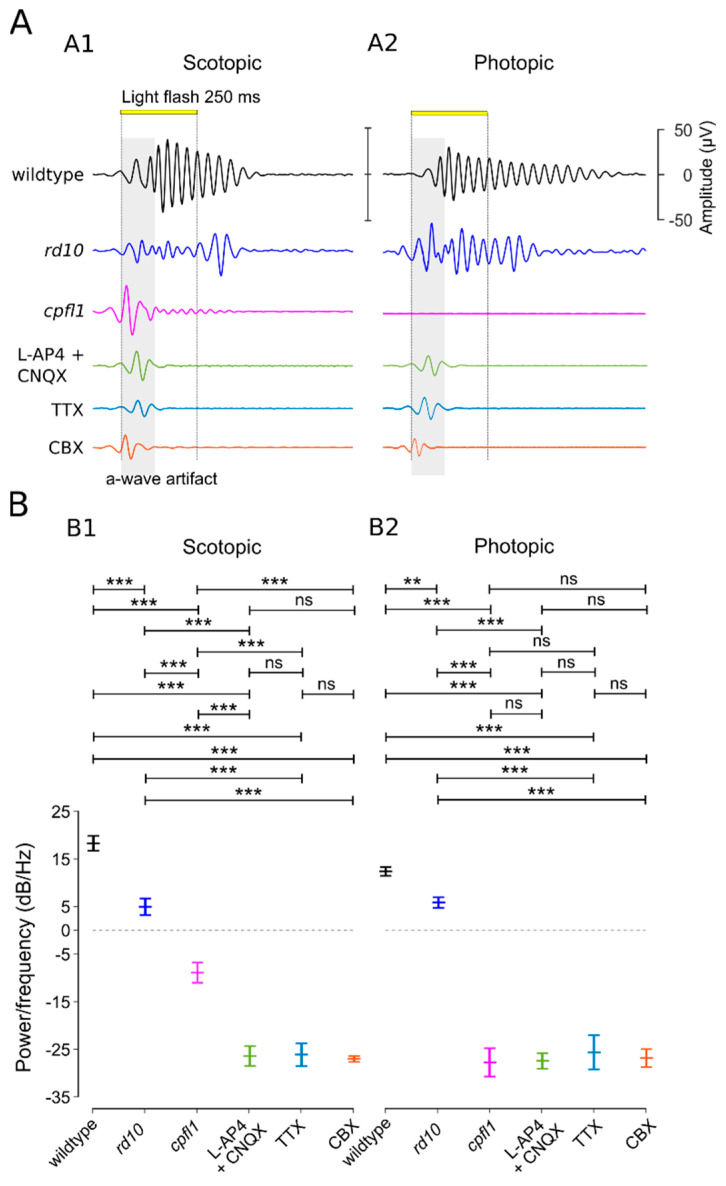
Analysis of oscillatory potentials in the µERG b-wave. (**A**) Representative traces of the oscillatory potentials (OPs) under (**A1**) scotopic and (**A2**) photopic conditions (band-pass filtered 17–200 Hz to avoid a-wave artefacts). Animals used: wild-type mouse, retinal degeneration-10 (*rd10*) mouse, and cone photoreceptor function loss (*cpfl1*) mouse. Drug applications were performed on wild-type mice, using either a combination of ionotropic glutamate receptor blocker CNQX and metabotropic glutamate receptor agonist L-AP4 (green), the voltage-gated sodium channel blocker TTX (blue), or the gap-junction blocker CBX (orange). (**B**) Statistical evaluation of OPs (power/frequency (dB/Hz) at the mean frequency (scotopic (**B1**): 36.52 ± 2.99 Hz; photopic (**B2**): 38.17 ± 2.62 Hz)). For each treatment condition, a separate wild-type retina was used (for each condition, *n* = 5 retinas/recording; each recording averaged from 59 electrodes). Statistical significance was assessed using one-way ANOVA followed by Dunnett’s multiple comparisons test (***: *p* < 0.001; **: *p* < 0.1; ns: not significant). Error bars indicate mean ± SEM.

The rod BC pathway in the inner retina does not connect directly to GCs but rather relies on ACs in the cone-BC pathway to connect to GCs (cf. Figure 1A, reviewed in [54]). Hence, the voltage-gated sodium channel blocker TTX was applied to block the signalling of ACs and GCs [11,31,55,56]. In the presence of TTX, OPs and GC responses were completely absent without affecting the photoreceptor responses (representative traces: Figure 3(A2,B2); statistical evaluation: Figure 3(A3,B3)), suggesting an AC-mediated synchronous drive of GCs. Disconnecting the gap-junction-coupled ACs (AC–AC) via the gap-junction blocker CBX eliminated OPs and synchronized GC responses, again without affecting a-wave amplitudes (representative traces: Figure 3(A2,B2); statistical evaluation: Figure 3(A3,B3); OP analysis: Figure 4). However, the block of gap-junctional coupling produced a GC spike pattern that no longer showed the characteristic oscillations but instead looked like a retinal ON–OFF response (Figure 3(A2,B2)). Overall, the data suggested that the OPs generated by ACs were dependent on gap-junctional electric coupling between ACs. This coupling may serve to synchronize GC oscillations.

## 4. Discussion

Developing new drugs for the treatment of retinal diseases requires extensive and thorough pre-clinical testing, which typically involves ERG recordings performed on in vivo animal models for retinal degeneration [21,57]. To help investigate drug effects on visual function as early as possible in translational research, we established a new µERG method that provides retinal function data comparable to in vivo ERG data, even at the level of in vitro testing. This new method allows us to simultaneously capture the light-evoked retinal activity originating from the various retinal layers and cells, notably from first-order neurons (photoreceptors), second-order neurons (BC and AC), and third-order neurons (GC). Although the latter cells are notoriously difficult to record using conventional ERG setups, the significantly higher temporal resolution of the µERG affords unprecedented insights into inner retinal signal processing. Together, these features highlight the unique capabilities of the µERG approach, which in addition, enables a reduction in in vivo experiments, facilitating the implementation of 3R principles in retinal drug development.

### 4.1. Ex Vivo µERG Recordings of Retinal Explants

The recordings obtained using the µERG technique on retinal explants largely match the signals seen in in vivo ERG, including those of wild-type and retinal degeneration mutants [21,45,47], and reflect all the characteristic features of neuroretinal signals [5]. µERG recordings under scotopic and photopic conditions allow the discrimination of rod and cone function, validated by the respective models for rod and cone dysfunction. The temporal and spatial resolution of this new technique is vastly superior to the conventional ERG technique. Our data were recorded at a very high sample rate of 25 kHz, which is in contrast with the atypical sample rate of 1 kHz or less [2], and were filtered at a broader range. This allowed for the first time to register the OPs with details not seen in previous studies [42,44,58].

Conventional in vivo ERG setups typically employ a 20 ms light flash [21,22]. Initially, we tested 500 ms light flashes, as proposed by [42], but found the 250 ms flash duration to be sufficient to achieve a maximum a-wave deflection and account for the subsequent GC responses (ON, OFF, and ON–OFF types), which may last for up to ~500 ms [49].

We additionally recorded the GC activity, allowing for a precise temporal correlation of the retinal signal from the photoreceptors to the GC responses. Clustering the GC responses into the three commonly known major functional types (ON = 45%, OFF = 20%, and ON–OFF = 35%) was consistent with previous findings [49]. However, the presentation of the recorded GC activity in a histogram format (integrated over all 59 electrodes) revealed a phase-locked, correlated oscillatory activity between OPs and GCs. Moreover, the spike responses can serve as an additional reliable indicator to verify light-correlated responses, particularly when the a-wave of the recorded µERG is very small, for example, in the rod domain at very low light intensities or in the cone domain.

Because of the long interval necessary for the complete dark adaptation of rods, the repeated application of the complete µERG protocol in the same explant is not recommended. Ex vivo dark adaptation for 12 h after one cycle of µERG recording, as usually performed in vivo, would not yield comparable results in terms of responsiveness to lower light intensities and overall response magnitude. It is conceivable that the explant’s thinner RPE layer requires more time to regenerate the photoreceptors’ rhodopsin content or that the used ACSF medium may not be as nutritious as a complete cell-culturing medium [57] for long-term applications.

### 4.2. Modulation of µERG Responses by Amacrine Cells

The importance of OPs for clinical investigations has been emphasised on many occasions [32,35,36,38,39]. However, in retinal physiology, the origin of OPs observed in ERG recordings has not yet been conclusively demonstrated. Since OPs may indicate specific functional states, their analysis and understanding are highly valuable, particularly for retinal disease diagnosis.

Regarding retinal pathologies, rod- and cone-driven OPs have been investigated extensively [50]. Recent studies have established that rod and cone coupling in the outer retina modulates the photopic ERG response [40]. Our µERG recordings of rod and cone mutants are in line with these findings and show OP alterations that are more marked in the *cpfl1* retina with cone dysfunction than in the *rd10* mouse model with rod dystrophy. This suggests an overall cone-pathway-dominated shaping of the OPs in the µERG.

The rod and cone pathways contact each other in both the outer and the inner retina. Hence, the question arises as to which relay node modulates the OPs. In the mammalian outer retina, the gap-junctional rod–cone coupling via connexin-36 (Cx36) serves as a secondary pathway for the rod signal bypassing the rod BC [59,60,61]. However, ERG recordings of Cx36^−/−^ mice still generated OPs [40]. Looking deeper into the inner retina, it has been proposed that the rod bipolar cell—AII amacrine cell reciprocal synapses are responsible for OP generation [62], the GCs contribute to the ERG [11,63], and the voltage-gated sodium channels [31] are involved in the modulation of OPs. In our experiments, the application of the voltage-gated sodium channel blocker TTX abolished the OPs and spike activity, which is plausible since voltage-gated sodium channels are known to be expressed in ACs and GCs [64]. However, the application of the gap-junction blocker CBX abolished OPs but not GC spike responses, resulting in an ON–OFF response pattern.

Our findings that the gap-junctional coupling of the AC–AC generates oscillatory activity in the retina are supported by previous studies from our own and other labs that investigated patch-clamp recordings [65,66,67,68]. In the retinal degeneration-1 (*rd1*) mouse model, the loss of rods and cones led to abnormal spontaneous oscillatory activity, which was silenced by gap-junction blockers but not by blockers of GABA, glutamate, or glycine receptors. The elimination of GABAergic suppression led to increased chaotic retinal activity [49,68]. Moreover, the presented µERG data of the mouse model with rod function loss (*rd10*) exhibited robust OPs, stemming from cone-pathway activation, whereas the mouse models with cone-function loss (*cpfl1*) showed poor OPs via rod-pathway activation. Hence, our µERG data support the idea that the OPs are shaped predominantly by the cone pathway [40] rather than by the rod bipolar cell—AII amacrine cell reciprocal synapses [62]. Overall, our data demonstrated that the OPs originate in the inner retina and rely on the gap-junction coupling of ACs. The vast gap-junctionally-coupled network of different amacrine cell types promotes an “oscillatory swing” of the signal through the network, shaping the Ops that consist of several wavelets.

## 5. Conclusions

The advanced µERG recording approach presented in our work introduces a sophisticated methodology to measure the function of both the outer and inner retina with an unprecedented temporal and spatial resolution. Notably, the possibility to relate GC activity to outer retina function creates opportunities for functional investigations not previously available in conventional ERG. Moreover, the high level of detail revealed by the µERG technique enabled us to show that OPs originate in the gap-junctionally-coupled AC network and likely contribute to the synchronization of GC activity. A better understanding of OPs may in the future be used to improve the non-invasive diagnosis of retinal diseases via conventional ERG. Finally, with some adaptations, the technique established here may also be applicable to investigations of other multi-layered neuronal tissues, such as stem cell-based retinal organoids [69] or brain organoids [70].

## Data Availability

The data presented in this study are available in the article.

## Abbreviations

The following abbreviations are used in this manuscript:

ERG	electroretinogram
µERG	micro-ERG
GC	ganglion cell
AC	amacrine cell
OP	oscillatory potentials
BC	bipolar cells
MEA	multi-electrode array
*cpfl1*	B6.CXB1-*Pde6c^cpfl^*^1*/J*^
*rd10*	C57BL/6J-*Pde6b^rd^*^10^
ACSF	artificial cerebrospinal fluid
mM	milli molar
L-AP4	L-2-amino-4-phosphonobutyric acid
NBQX	2,3-Dioxo-6-nitro-1,2,3,4-tetrahydro-benzo[f] quinoxaline-7-sulfonamide
TTX	tetrodotoxin
CBX	carbenoxolone
kHz	kilo hertz
GJ	gap junction
mGluR6	metabotropic glutamate receptors
iGluR	inotropic glutamate receptors
VGNaC	voltage-gated sodium channels
R	rod
C	cone
RB	rod bipolar cell
H	horizontal cell
CB OFF	OFF-type cone bipolar cell
CB ON	ON-type cone bipolar cell
AII	amacrine cell-type AII
µV	microvolt
Hz	hertz
kHz	kilo hertz
Log	logarithm of base 10
SEM	standard error mean
ns	not significant
ANOVA	analysis of variance
P	statistical significance value
ctr	control
dB	decibel
GABA	gamma-aminobutyric acid
